# When natural mutants do not fit our expectations: the intriguing case of patients with *XRCC4* mutations revealed by whole-exome sequencing

**DOI:** 10.15252/emmm.201505307

**Published:** 2015-05-11

**Authors:** Jean-Pierre de Villartay

**Affiliations:** 1 INSERM UMR1163, Laboratory of Genome Dynamics in the Immune System (DGSI)Paris, France; 2 Paris Descartes University-Sorbonne Paris Cité, Imagine InstituteParis, France

## Abstract

Mutations in the *XRCC4* gene have been recently identified through whole-exome sequencing (WES). While the overall clinical presentation of the patients (severe short stature, microcephaly, gonadal failure) generally conforms with what is expected for the defect of a critical non-homologous end-joining (NHEJ) DNA repair factor, the absence of consequence on the proper development of the immune system is rather surprising, given the role of NHEJ in V(D)J recombination. Several hypotheses can be envisioned to explain this discrepancy. Overall, these findings highlight the power of WES in identifying new molecular causes for human diseases while providing with new exciting scientific question to address.

See also: **L Bee *et al*** (July 2015)

Up to now, molecular medicine or the art of identifying deleterious, disease-causing mutations in genes relied on knowledge-based sequencing of a handful of candidates. At best, this approach was optimized by prior genetic studies (whole-genome association studies (WGAS) or whole-genome homozygosity mapping (WGHM)) to restrict the list of candidates in case of consanguineous families and/or large series of patients. The field has moved one step forward in recent time with the completion of the human genome sequence and the development of next-generation sequencing (NGS) of DNA covering all coding exons (whole-exome sequencing (WES)). Five studies, including the one by Bee *et al* (2015) in this issue, recently reported on mutations in the *XRCC4* gene in 12 human patients identified through WES (Fig1) (Gennery *et al*, 2014; Shaheen *et al*, 2014; de Bruin *et al*, 2015; Murray *et al*, 2015). Most of the cases presented with microcephalic primordial dwarfism (MPD) and gonadal failure. Early-onset metabolic syndrome or cardiomyopathies were also noticed in some patients. Was this the kind of clinical presentation one would have expected for XRCC4 deficiency?

**Figure 1:**
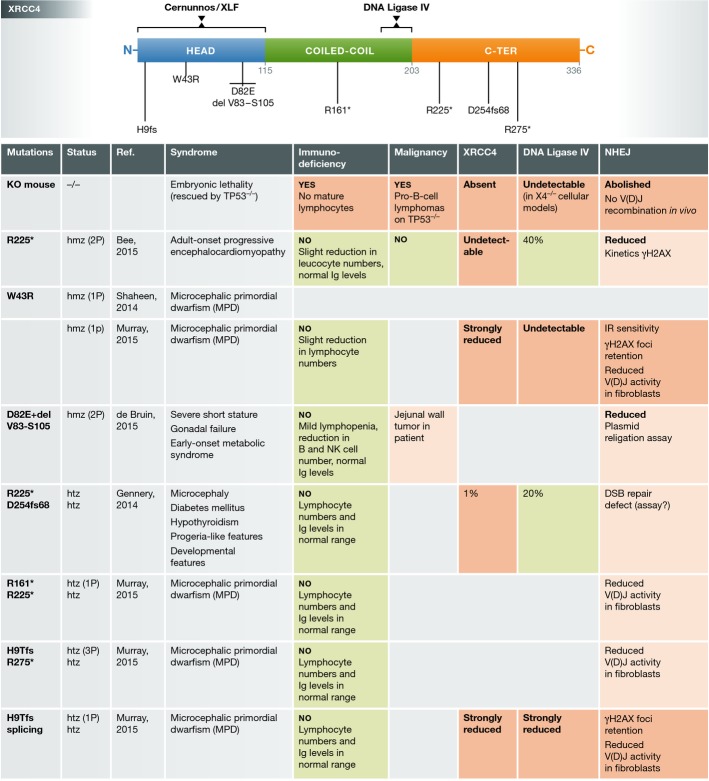
XRCC4 mutations in humans Top: Position of the XRCC4 mutations with respect to the 3 known domains of XRCC4. Regions of interaction of XRCC4 with Cernunnos/XLF and DNA ligase IV are also represented. Bottom: Summary of the published cases of XRCC4 deficiency in humans. Phenotype of XRCC4 KO mice is included for comparison.

XRCC4 stands for X-ray repair cross-complementing protein 4, the mutation of which results in the increased sensitivity to ionizing radiations of the Chinese hamster ovary (CHO) cell line XR-1 (Li *et al*, 1995). It represents one of the core factors of the non-homologous end-joining (NHEJ) DNA repair pathway. The NHEJ is one of the two main DNA repair pathways (with the homologous recombination) that account for the resolution of DNA double-strand breaks (DNA DSB). Very schematically, the NHEJ apparatus is composed of seven known core factors: Ku70/Ku80/DNA-PKcs that constitute the DNAPK complex, the DNA endo/exonuclease Artemis, and the XRCC4/Cernunnos–XLF/DNA ligase IV complex (see van Gent and van der Burg, 2007 for review). XRCC4 is thought to be essential since *XRCC4* KO mice die during embryogenesis owing to the massive apoptosis of post-mitotic neurons, a phenotype shared by DNA ligase IV KO but not the other NHEJ-deficient murine models. One has to keep in mind though that the absence of XRCC4 results in the destabilization and degradation of DNA ligase IV. It may well be that the dramatic phenotype of *XRCC4* KO mice results, in part, from the associated loss of DNA ligase IV. The embryonic lethality can be rescued by the introduction of TP53 defective alleles. The syndromic characteristics of the newly identified *XRCC4*-defective patients are globally conform (apart from the embryonic lethality) to the phenotype observed with various *XRCC4* conditional/rescued mice except for one major aspect: These patients do not suffer from any sign of immunodeficiency and their immune system develops normally, apart for a mild lymphopenia noted in some cases. This is a rather surprising observation given the critical role of the NHEJ pathway during V(D)J recombination in lymphocytes. V(D)J recombination is a DNA somatic rearrangement process exclusively confined to immature B and T lymphocytes, the function of which is to assemble gene segments that will encode for the highly diverse antigenic receptors (immunoglobulins and T-cell receptors) expressed by B and T cells. V(D)J recombination is initiated through the introduction of DNA DSB in Ig and TCR loci by the lymphoid-specific factors Rag1 and Rag2, followed by their NHEJ-mediated repair (see Schatz & Swanson, 2011 for review). One consequence of faulty V(D)J recombination, either in its initiation phase or during DNA repair, is the early arrest of B and T lymphocyte development, the lack of a functional adaptive immune system, and the ensuing severe combined immune deficiency (SCID) (de Villartay *et al*, 2003). Indeed, patients with V(D)J recombination deficiency present with T-B-NK+ SCID and die from severe infections in their first year of life in the absence of treatment such as hematopoietic stem cell transplantation. Likewise, TP53^−/−^ rescued *XRCC4* KO mice display a complete absence of mature lymphocyte development owing to their impaired V(D)J recombination, a trait accompanied by the onset of aggressive pro-B-cell lymphomas, revealing the role of XRCC4 as an important genome caretaker. For memory, the *XRCC4* gene was in fact identified through cDNA functional complementation of the V(D)J recombination deficiency of XR1 cells (Li *et al*, 1995).

How can one reconcile the critical function of NHEJ during V(D)J recombination and the apparent absent immune phenotype of XRCC4-defective patient, even though these patients sometimes present with very severe clinical manifestations? One explanation could be that the *XRCC4* mutations are hypomorphic, thus bypassing the suspected embryonic lethality and allowing V(D)J recombination to occur, leaving the immune system unaffected. Indeed, in some of the described patients, a significant level of DNA ligase IV expression is preserved in contrast to what happens with complete loss of function alleles. Nevertheless, hypomorphic mutations in the DNA ligase IV gene are often associated with impaired adaptive immunity as seen by the recurrent common childhood infections (Murray *et al*, 2014). When surveyed, these patients often display hypogammaglobulinemia and low B lymphocyte counts. Another possibility could be that the presence of one or more redundant factors can accommodate mutations in the *XRCC4* gene. Indeed, XRCC4 belongs to a family of structurally related proteins that also comprises Cernunnos/XLF. Two recent reports extended this family by adding the PAralog of XRCC4 and XLF (PAXX) factor, also known as C9orf142 (Ochi *et al*, 2015; Xing *et al*, 2015). PAXX, a direct interactor of Ku, is a *bona fide* NHEJ factor that was shown to function redundantly with Cernunnos/XLF in particular situations of DNA damage response. Whether this redundancy also applies to XRCC4 is an interesting issue to rise. A last proposal would be that, once XRCC4 ensues its function of DNA ligase IV stabilization (most of the mutations described spare expression of DNA ligase IV to some extent), it becomes dispensable during immune system development while still required in other DNA damage response situations such as in the brain. There is an interesting precedent provided by the analysis of Cernunnos/XLF-deficient mice (Vera *et al*, 2013). Although Cernunnos/XLF deficiency results in severely impaired DNA DSB repair in fibroblasts, the V(D)J recombination is not overwhelmingly affected in these animals and the reduced T-cell number is mostly the consequence of a diminished thymocyte survival. It was proposed that the filament formed by XRCC4 and Cernunnos/XLF (Ropars *et al*, 2011), critical to tether DNA ends during DNA DSB repair following genotoxic agents, may be “backuped” in lymphoid cells by the DNA end synapse formed by the Rag1 and Rag2 post-cleavage complex (PCC) once V(D)J recombination has been initiated (Vera *et al*, 2013). Indeed, Cernunnos/XLF becomes critical for V(D)J recombination and lymphocyte development when the PCC is destabilized (unpublished observation). With this in mind, some of the *XRCC4* mutations reported in humans become very interesting as they affect residues localized in the head domain, the region of interaction with Cernunnos/XLF. It would be of particular interest to analyze the impact of these mutations on the formation of the XRCC4–Cernunnos/XLF filament.

In summary, the recent identification of *XRCC4* mutations in humans is emblematic of the strength of the newly developed exome-sequencing-driven molecular medicine not only in revealing unexpected structure/function relationships for given factors but also in providing basic science with new enigma to resolve.
